# Melanoma: Multiple Presentations of an Invisible Illness

**DOI:** 10.7759/cureus.47465

**Published:** 2023-10-22

**Authors:** Inês Santos, Maria Leonor Guia Lopes, Catarina I Cabral, José Cidade, João Furtado

**Affiliations:** 1 Internal Medicine, Hospital Egas Moniz, Lisboa, PRT; 2 Endocrinology, Hospital Egas Moniz, Lisboa, PRT

**Keywords:** subcutaneous metastasis, lymph node metastasis, muscle metastasis, occult primary melanoma, malignant melanoma metastasis

## Abstract

Melanoma can spread to any organ of the body. The most affected sites are the skin and subcutaneous tissue, lymph nodes, lungs, liver, brain, bone, and intestine. Early diagnosis is crucial to prompt treatment. Although the incidence of melanoma is rising, novel treatment options are being developed, enabling a better prognosis. The authors present a rare case of metastatic melanoma affecting the muscle, lymph nodes, and subcutaneous tissue. The patient complained of redness and swelling of the right thigh and inguinal region, red, painful lumps on her chest wall, and pain in the left upper abdominal quadrant. A CT of the thorax, abdomen, and pelvis was performed, and surgical excision of the left thoracic mass led to the diagnosis of metastatic melanoma. However, no primary lesion was found despite extensive investigation. The unusual presentation of muscular metastasis heralds a poor prognosis. This case highlights the difficulty of diagnosing patients with rare presentations of a rather frequent disease.

## Introduction

Melanoma is a type of skin cancer that can spread to any body organ [1]. The most affected sites are the skin and subcutaneous tissue, lymph nodes, lungs, liver, brain, bone, and intestine [1]. Muscle metastases are unusual and herald a poor prognosis [2]. It can be a presenting symptom of melanoma [1]. New therapeutic targets are emerging, and prompt treatment is essential to prevent progression [1]. Considering that there are very few cases described in the literature, the authors report a rare case of muscular metastasis as a presenting symptom of melanoma.

## Case presentation

The authors describe the case of a 64-year-old Caucasian woman. Her past medical history included biclonal IgG and IgA gammopathy and lumbar hernia. Her past surgeries included an excision of a benign thyroid cyst and a hysterectomy due to myoma. She did not take any medication.

The patient presented to the emergency department with a 24-hour history of pain, redness, and swelling of the right thigh and inguinal region. She also complained of red, painful lumps on her chest wall, as well as night sweats, anorexia, and a 3-kg weight loss over the last three weeks. She denied any other symptoms. The patient was previously treated with antibiotics but showed no improvement.

On physical examination, she was alert and fully oriented. Her vital signs were stable, and she was afebrile. Chest and abdominal examination showed multiple painful masses on her left upper abdominal quadrant, left chest wall, right chest wall, and parasternal region. She also had firm fixed lymph nodes on her right inguinal, left axillary, and left cervical regions. Her limb examination showed redness and tenderness on her inner right thigh.

**Figure 1:**
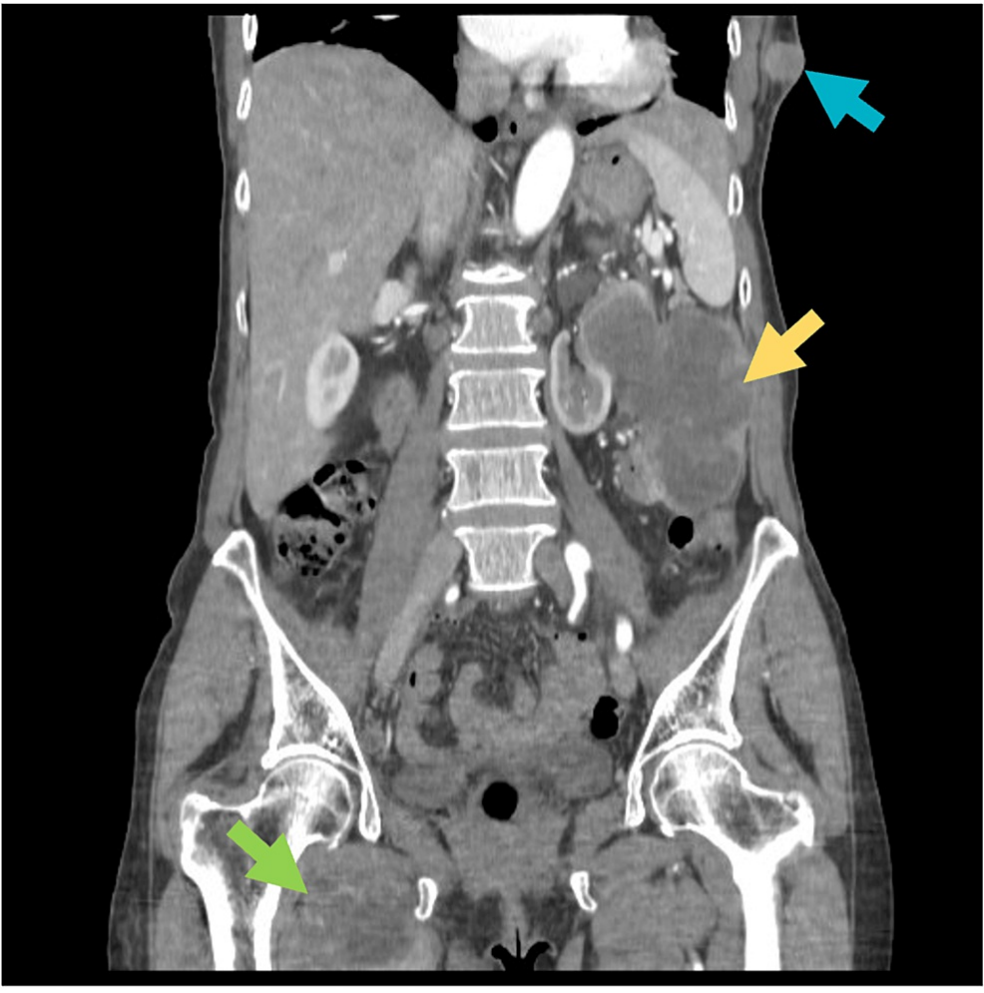
Masses on CT scan The blue arrow shows the left thoracic mass, the yellow arrow shows the left retroperitoneal mass, and the green arrow shows the inner right thigh mass.

Blood analysis showed slight anemia (hemoglobin 11.6 g/dL), leukocytosis (19900 cells/uL), and increased C-reactive protein (6.9 mg/dL). Human immunodeficiency virus serology and blood cultures were negative. A CT of the thorax, abdomen, and pelvis was performed, which revealed a 10-cm left retroperitoneal mass with a necrotic center, as well as smaller thoracic and inner right thigh masses, and left paraaortic, right retroperitoneal, and right inguinal adenopathy conglomerates (Figure 1). She was treated with a five-day course of clindamycin but showed no clinical improvement.

Due to the possibility of malignancy, the patient was admitted for further investigation. Carcinoembryonic antigen, cancer antigen 15-3, and alpha-fetoprotein levels were normal. A surgical excision of the left thoracic mass was performed. Histology showed metastasis from melanoma, with lymphatic and perineural invasion, and positivity for HMB45, S100, melan-A, and SOX10. BRAF mutation was negative. Her microbiological examination was also negative.

The diagnosis of metastatic melanoma was made, with muscle, soft tissue, and lymph node involvement. No primary cutaneous or mucous lesion was identified. Brain MRI did not show any lesions. PET also did not show any additional involvement.

Hence, she was referred for immunotherapy palliative treatment with an anti-programmed cell death-1 (PD-1) agent and palliative radiotherapy for the muscular metastases. However, she experienced rapid clinical deterioration and died before starting targeted therapy.

## Discussion

Melanoma arises from the malignant transformation of melanocytes [3]. Melanocytes originate from the neural crest and migrate to the skin, gastrointestinal tract, and brain; therefore, these are the most common melanoma sites [3]. Melanoma cells can spread radially and vertically [3]. Vertical growth can lead to metastases to other body parts [3]. The skin and subcutaneous tissue are the most common sites, followed by the lungs, liver, bones, and brain [1]. Muscle metastasis is rare and usually associated with poor prognoses [2].

The median age of melanoma diagnosis is 57 years [3]. Its incidence is rapidly increasing, and it is the fifth and seventh most common malignancy in men and women (5% and 4% of malignant neoplasms), respectively [3]. Although less common than basal and squamous cell carcinoma, melanoma is the most lethal cutaneous malignancy and the third most lethal neoplasm overall [1,3]. Stage IV patients experience a grim 10% five-year survival rate [3]. Mucosal and ocular melanoma have the worst prognosis [1].

The risk factors for melanoma include family history (5-12% of cases), certain physical traits, such as fair skin, red hair, blue eyes, presence of freckles or melanocytic nevi, conditions such as immunosuppression and dysplastic nevus syndrome, excessive sun exposure, and lower socioeconomic status [3,4].

Diagnosis is made by histologic evaluation of the lesion [3]. Full-thickness excisional biopsy is usually required [3]. Histopathology findings include lesions with ill-defined borders, asymmetrical proliferation of atypical melanocytes, loss of melanocyte differentiation, presence of pagetoid melanocytes, and lymphocytic response [1]. Ulceration is usually associated with poor prognosis. The depth of invasion is the most important prognostic factor [1].

Initial workup includes a complete blood count, serum biochemistry panel, and chest radiography [3]. Brain MRI, CT of the chest, abdomen, and pelvis, and whole-body PET can be used to exclude metastatic disease [3]. Melanoma staging is based on the TNM system by the American Joint Committee on Cancer (tumor thickness with or without ulceration, nodal involvement, and metastasis) [1].

Treatment of early-stage melanoma includes primary lesion excision and sentinel lymph node biopsy or elective node dissection [3]. However, a negative lymph node does not exclude metastasis and does not affect the overall prognosis [1,3]. Advanced-stage melanoma treatment includes adjuvant therapies, such as chemotherapy, immune checkpoint inhibitors, and targeted therapy based on the BRAF genotype [1]. Wild-type BRAF melanoma can be treated with anti-PD-1 agents, and BRAF-mutant melanoma can be treated with BRAF/MEK inhibitor combination therapy [1]. These novel treatment agents have improved the outcomes of some patients [1]. Metastatic diseases, such as visceral, bone, and central nervous involvement, can be treated with radiation therapy [1]. Complications of melanoma include secondary infection, scarring, lymphedema, and local recurrence [3].

## Conclusions

We report this case due to the unusual presentation of muscular metastasis as a presenting symptom of melanoma. In this patient, no primary lesion was found despite extensive investigation. This highlights the difficulty of diagnosing patients with rare presentations of a rather frequent disease. Metastatic disease at presentation, particularly muscular involvement, usually heralds a poor prognosis, as shown in this case, in which the patient died before starting targeted therapy.
